# CVD growth of large-area monolayer WS_2_ film on sapphire through tuning substrate environment and its application for high-sensitive strain sensor

**DOI:** 10.1186/s11671-023-03782-z

**Published:** 2023-02-16

**Authors:** Weihuang Yang, Yuanbin Mu, Xiangshuo Chen, Ningjing Jin, Jiahao Song, Jiajun Chen, Linxi Dong, Chaoran Liu, Weipeng Xuan, Changjie Zhou, Chunxiao Cong, Jingzhi Shang, Silin He, Gaofeng Wang, Jing Li

**Affiliations:** 1 Engineering Research Center of Smart Microsensors and Microsystems, Ministry of Education, College of Electronics and Information, Hangzhou Dianzi University, Hangzhou, 310018 China; 2 State Key Laboratory of ASIC and System, School of Information Science and Technology, Fudan University, Shanghai, 200433 China; 3 Department of Physics, School of Science, Jimei University, Xiamen, 361021 China; 4 High Tech Center for New Materials, Novel Devices and Cutting Edge Manufacturing, Yiwu Research Institute of Fudan University, Chengbei Road, Yiwu City, 322000 Zhejiang China; 5 Institute of Flexible Electronics (IFE), Northwestern Polytechnical University (NPU), 1 Dongxiang Road, Chang’an District, Xi’an, 710129 China; 6 Collaborative Innovation Center for Optoelectronic Semiconductors and Efficient Devices, Pen-Tung Sah Institute of Micro-Nano Science and Technology, Xiamen, 361005 China

**Keywords:** 2D materials, WS_2_, CVD, COMSOL, Strain sensor

## Abstract

Large-area, continuous monolayer WS_2_ exhibits great potential for future micro-nanodevice applications due to its special electrical properties and mechanical flexibility. In this work, the front opening quartz boat is used to increase the amount of sulfur (S) vapor under the sapphire substrate, which is critical for achieving large-area films during the chemical vapor deposition processes. COMSOL simulations reveal that the front opening quartz boat will significantly introduce gas distribute under the sapphire substrate. Moreover, the gas velocity and height of substrate away from the tube bottom will also affect the substrate temperature. By carefully optimizing the gas velocity, temperature, and height of substrate away from the tube bottom, a large-scale continues monolayered WS_2_ film was achieved. Field-effect transistor based on the as-grown monolayer WS_2_ showed a mobility of 3.76 cm^2^V^−1^ s^−1^ and ON/OFF ratio of 10^6^. In addition, a flexible WS_2_/PEN strain sensor with a gauge factor of 306 was fabricated, showing great potential for applications in wearable biosensors, health monitoring, and human–computer interaction.

**Supplementary Information:**

The online version contains supplementary material available at 10.1186/s11671-023-03782-z.

## Introduction

In recent years, two-dimensional (2D) materials with atomic thickness have attracted great interest due to their excellent electrical and mechanical properties and have been successfully applied in electronics, sensing, energy, and other fields [1–3]. Graphene, as a 2D material, is prevented from being used in logic electronics and FETs due to its inherent zero band gap and chemical inertness. In contrast, transition metal dichalcogenides (TMDs) [4, 5], such as WS_2_ and MoS_2_, compensate for the shortcomings of graphene in these applications due to the special sandwich atomic structure arrangement (X–W–X) and physical properties and show emerging properties when reduced to monolayer [6]. WS_2_ and MoS_2_ have many similar excellent properties, such as the adjustable band gap, coupled spin and valley physics [7–10], and band structure tunability with strain [11]. These properties make monolayer WS_2_ and MoS_2_ promising candidates for electronics, photonics, and valley electronics [12]. Furthermore, WS_2_ and MoS_2_ have been used to form flexible/wearable electronic sensors for monitoring physiological health signals due to their excellent mechanical flexibility and perfect conformability to non-flat surfaces of the human body [13, 14]. Therefore, how to grow large-area continuous TMDs films has attracted significant interest, which is a prerequisite for their widespread use. Many attempts have been made to prepare TMDs such as ionized jet deposition [15], mechanical exfoliation [16, 17], CVD using WO_3_ and MoO_3_ with sulfur powder, etc. [18–20]. Compared to the lateral dimensions of flakes synthesized by exfoliation methods, which limit their application in large-scale electronics [21], CVD is considered as a promising method to fabricate large-scale and continuous TMDs films [22]. For a facile one-step metal–organic CVD (MOCVD) of TMDs, although it is desirable to use a single-source precursor containing the corresponding metal and required sulfur in the ligand sphere, the thermal properties of most of the proposed precursors are insufficient for MOCVD [23]. There are also some reports on the synthesis of WS_2_ nanosheets on SiO_2_ by atomic layer deposition (ALD) [24]. However, not only does this approach require additional annealing steps with H_2_ and H_2_S to further convert the oxide precursors to WS_2_ [25, 26], but the high toxicity of H_2_S is also an issue. Recently, it has been reported that modifying the miscut orientation toward the A axis (C/A) of sapphire can finally achieve large-area continuous films with more than 99% unidirectional alignment [27]. However, when growing WS_2_ or MoS_2_ crystals on sapphire in this miscut orientation, the substrate needs to be annealed in O_2_ for 2 h first, which may increase the preparation time.

Herein, we present a simple method to grow large-area and continuous monolayer WS_2_ films on the sapphire substrate by CVD reaction between WO_3_ and sulfur using the front opening quartz boat. In previous reports, little attention has been paid to the gas flow distribution under the sapphire substrate and the height of the sapphire substrate during the preparation process. To obtain large-area continuous WS_2_ films, computational fluid dynamics and thermodynamics simulations were first carried out using finite element analysis software to investigate the gas and temperature distribution around the substrates. The results of the simulations provided us with a direction to optimize the growth conditions. Then, we changed the quartz boat, which means cutting the front end of the quartz boat, to make the gas flow uniformly and steadily throughout the growth chamber, and controlled the height of the sapphire substrate into the furnace by making quartz bases with different widths. The nucleation and coverage of WS_2_ crystal were also controlled by adjusting the growth temperature and the gas velocity. Raman spectroscopy, photoluminescence (PL), and optical micrograph were performed to characterize the surface morphology, domain size, and crystal quality of the WS_2_ samples. Meanwhile, the electrical properties of the as-grown monolayer WS_2_ films were also studied by fabricating and characterizing a top-gate FET. Higher field-effect electron mobility and switch ratio were observed than those previously reported [19, 28]. In addition, a transparent WS_2_ strain sensor with a GF of approximately 306 was fabricated on PEN flexible substrate, which also demonstrated the excellent electronic properties and mechanical flexibility of the as-grown WS_2_ films.

## Experiments

### Synthesis of monolayer WS_2_ film

Large-scale continuous monolayer WS_2_ films were prepared at atmospheric pressure in a CVD system with two separately controlled heating zones (AnHui BEQ Equipment Technology Co., Ltd.). Before the experiment, 20 mg of high-purity NaCl crystals (Macklin, 99.99%) was dissolved in 100 ml of deionized (DI) water to obtain a NaCl solution (as the growth promoter [29]). Sapphire (0001) substrates were cut to 0.5 × 1.8 cm in size and soaked in the prepared NaCl solution for 10 min. The reason for choosing a sapphire substrate here is that sapphire is hexagonal and compatible with the symmetry of the WS_2_ lattice, making it easier to grow monolayer WS_2_ films [30, 31]. Then, the front opening quartz boat carrying 200 mg sulfur powder (Aladdin, > 99.99%) as precursor was placed in a low-temperature furnace for sublimation at 150 °C, while 75 mg WO_3_ powder (Sigma-Aldrich, > 99.99%) as W source was loaded into another front opening quartz boat that carried by a 3.5-cm flat quartz in a high-temperature furnace heated at 975 °C. Next, the sapphire substrates were placed on the top of the WO_3_ powder (face down) with an interval of 1 mm apart. When the tubular furnace was evacuated to 10^–1^ Pa, high-purity Ar (99.999%) was introduced into the tube furnace to return to the atmospheric pressure, and it is important to note that Ar is kept in the tube furnace during the growth period to maintain an oxygen-free growth environment. After that, the high-temperature furnace began to heat up at a rate of 30–40 °C/min. Ten minutes before the high-temperature furnace reached the predetermined growth temperature, the preheating furnace began to heat up at a rate of 20 °C/min. The whole growth cycle lasted about 6 h, and then, the grown WS_2_ films were obtained on the sapphire substrates.

### Transfer of monolayer WS_2_ film and device fabrication

The as-grown WS_2_ films were transferred from the sapphire substrate to a new SiO_2_/Si (*p*-doped Si substrate with 300 nm SiO_2_) substrate or PEN substrate by a wet transfer method using DI water. The samples were first spin-coated with a layer of PMMA using a homogenizer (step 1: 500 rpm for 10 s; step 2: 2000 rpm for 60 s) and then baked on a hot plate at 175 °C for 2–3 min. The edges of the sapphire substrate were ground with a knife to expose the sapphire edges to DI water. Due to the hydrophobicity of WS_2_ material and hydrophilicity of the sapphire, the water surface tension can make the PMMA/WS_2_ float on the DI water. Next, the PMMA/WS_2_ films were picked up with a new SiO_2_/Si or PEN substrate and baked on a hot plate at 80 °C for 30 min to promote the boding between them. The as-transferred WS_2_/SiO_2_/Si samples were eventually obtained by removing PMMA in acetone solution at room temperature for 2 h. Then, the samples were further spin-coated with the LOR/S1818 photoresist and exposed using designed source and drain electrode pattern by laser direct writing lithography (Durham Microwriter ML3), followed by thermal evaporation deposition of Cr/Au (5/70 nm) and removal of the photoresist with acetone. The defined width and length of the channel are both 5 μm. For the WS_2_/PEN strain sensor, the surface of the PEN substrate requires a hydrophilic treatment first with a UV-ozone cleaner (CC1250GF-TC, Shanghai CHI Instrument CO., LTD, China) for 40 min. Then, the strain sensor can be completed by printing interdigital electrodes on the PEN substrate using a microelectronic flexible printer (Scientific 3A, Portronics, China) and transferring the WS_2_ films to the PEN substrate as described above.

### Optical and electrical characteristics measurements

The Raman and PL measurements were carried out by a confocal microscope alpha300 R (WITec GmbH, Germany) under excitation of 532 nm laser through a grating spectrometer with a thermoelectrically cooled detector. The I–V characteristic of the FET transmission and output was performed at room temperature by using a semiconductor analysis system (Agilent B1500) in combination with an on-board probe station. Besides, the strain sensor responses at different strains were measured directly in air by digital source-meter (Keithley 2450) combined with a modified Vernier caliper.

## Results and discussion

**Fig. 1:**
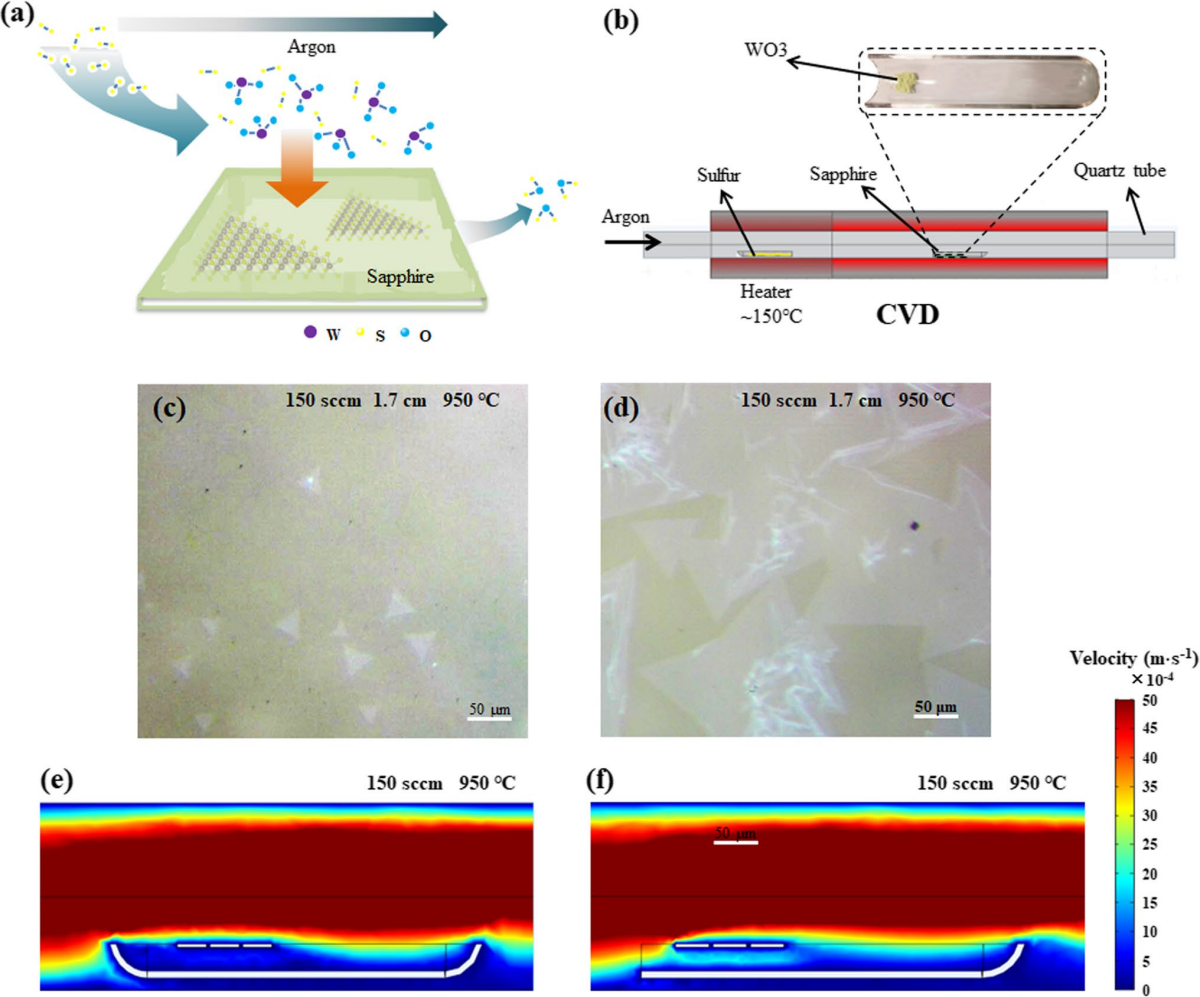
Schematics of **a** the related chemical reaction and **b** CVD system (the inset is the scenery of the sapphire and WO_3_ placed on the quartz boat). **c**–**d** The optical images of as-grown WS_2_ on sapphire substrates with traditional conventional quartz boat and front opening quartz boat, respectively. **e**–**f** COMSOL simulation of gas distribution of traditional quartz boat and front opening quartz under the same conditions, respectively

Figure 1a depicts the chemical reaction associated with the growth of WS_2_ during CVD growth. The volatile suboxide species WO_3−*x*_ is first formed through partial reduction of WO_3_ by sulfur vapor. Subsequently, WS_2_ is formed on sapphire by further sulfurization, as shown in the chemical reaction expression (1).

1 $$7{\text{S}} + 2{\text{WO}}_{3} \to 2{\text{WS}}_{2} + 3{\text{SO}}_{2}$$

A schematic of the CVD system is shown in Fig. 1b. The inset of Fig. 1b exhibits the scenery of the sapphire substrates and WO_3_ placed on the quartz boat. It is well known that WS_2_ growth is very sensitive to gas velocity and temperature. Therefore, both the speed of gas velocity and the temperature determine whether or not large-scale WS_2_ films can be obtained. An effective way to solve these problems is to optimize the appropriate gas velocity in the range of 100–200 sccm and the furnace growth temperature ranging from 850 to 950 °C. As shown in Fig. 1c, by using the conventional quartz boat without front opening, only a few small WS_2_ triangle domains with a size of about 32 μm appear in the central region of the sapphire substrates. In contrast, under the same conditions, more and larger WS_2_ triangles that merge into large scale can be achieved when using a quartz boat with front opening, as illustrated in Fig. 1d. To find out the reason for this result, the growth environment of WS_2_ films, especially the gas velocity and temperature distribution in the tube furnace, was simulated using COMSOL software. The structural model constructed is shown in Fig. S1, and more details about the simulations can be found in the supplementary material. Figure 1e depicts the distribution of gas velocity in the tube furnace when growing WS_2_ thin films with conventional quartz boat. It can be clearly found that the argon carrier gas is less distributed under the sapphire substrates due to the obstruction of the front of the conventional quartz boat. Hence, a small amount of sulfur vapor carried by the argon carrier gas is delivered to the bottom of the sapphire substrates and reacts with the vapor of WO_3_. Besides, the front end of the traditional quartz boat can obstruct and cause gas in the chamber to form turbulence, which makes the formation of random nucleation sites more likely and leads to unstable growth conditions [32]. In order to increase the sulfur vapor under the sapphire substrates, its front was cut off to form an opening, which was expected to facilitate the transport of sulfur vapor. As shown in Fig. 1f, because of the absence of obstruction, the front opening quartz boat does not block the flow of argon carrier gas, which indeed boosts the gas to flow through the bottom of the sapphire substrates (namely the smooth surface), and huge amount of sulfur vapor is transported to the smooth surface of the sapphire substrates and react with the vapor of WO_3_ to form more stable WS_2_ crystal nuclei. All the following experiments about the optimization of parameters such as temperature, air velocity and sapphire substrate height are based on the front opening quartz boat.

**Fig. 2:**
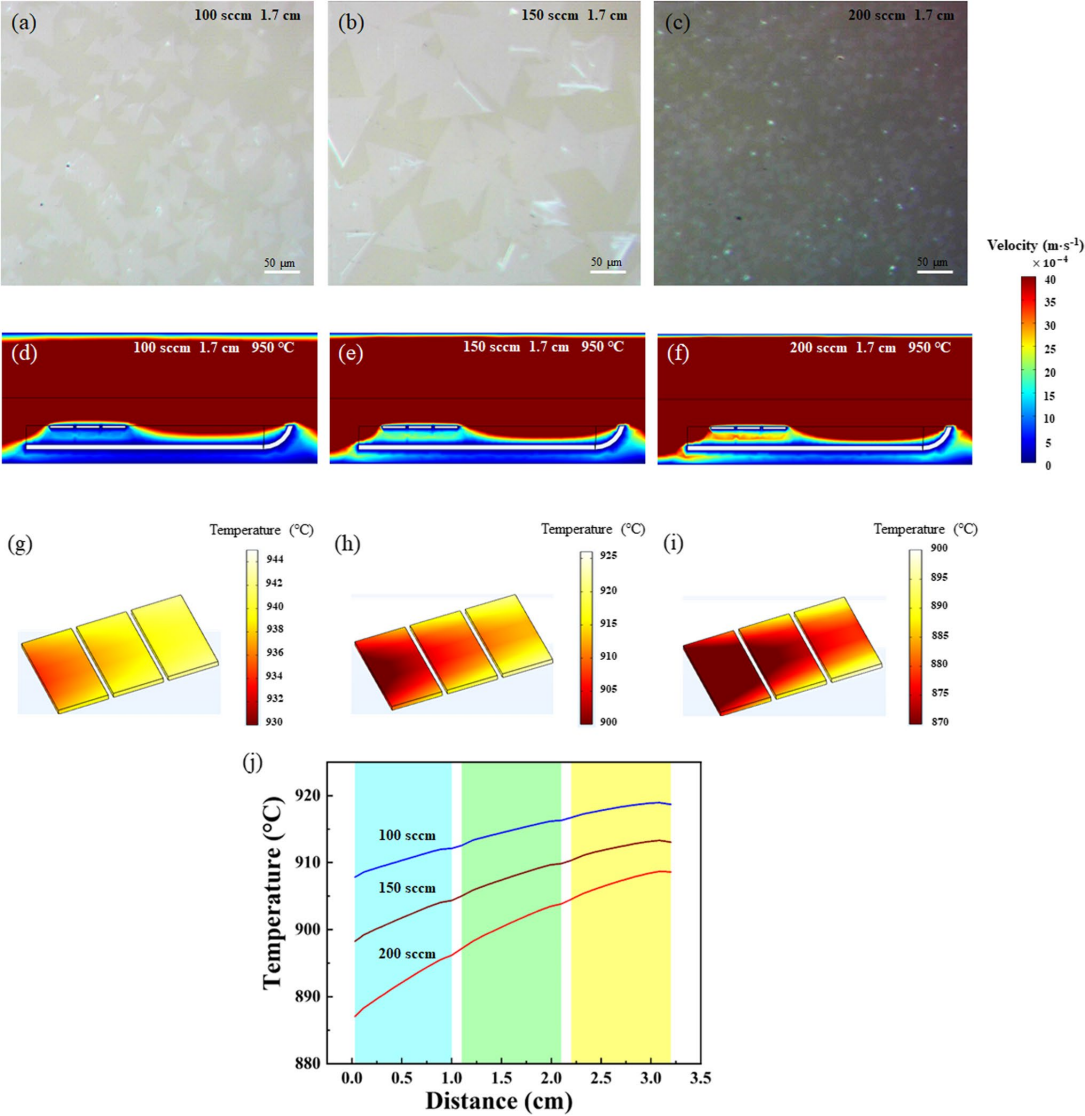
**a**–**c** The optical images of WS_2_ samples grown on sapphire substrates at gas velocity of 100, 150, 200 sccm, respectively. COMSOL simulation of **d**–**f** gas and **g**–**i** temperature distribution around the three sapphire substrates in the front opening quartz at gas velocity of 100, 150, 200 sccm under fixed 950 °C, respectively. **j** The extracted temperature curves along the middle line of the three sapphire substrates

In order to optimize the most suitable gas velocity for WS_2_ growth with the front opening quartz boat, the influence of different gas velocities on the growth rate and coverage of WS_2_ film was investigated. The gas velocities were set to 100, 150 and 200 sccm, respectively, with a fixed temperature of 950 °C and a height of 1.7 cm of the sapphire substrate away from the tube bottom. The corresponding morphologies of the as-grown WS_2_ samples are shown in Fig. 2a–c, respectively. Obviously, the WS_2_ crystals evolve with the gas velocity. It is well known that a key to WS_2_ nucleation and growth lies in the concentration ratio of S atom to W atom [33]. If the carrier gas velocity is too low, the S atoms carried by the gas may condense on the quartz tube before reaching around the substrates and combining with WO_3_ molecules. While the concentration ratio of W to S does not reach a critical value, WS_2_ will not form and nucleate on the substrate. When the Ar velocity is set to 100 sccm (Fig. 2a), there are a large number of WS_2_ triangles, but the size is small (~ 35 μm), and some WS_2_ crystals are not regularly triangular. As the gas velocity is increased to 150 sccm, the WS_2_ crystals become larger with about 114 μm and partially merge into large scales (Fig. 2b). The increase in carrier gas velocity will provide sufficient kinetic energy for the W and S atoms, so as to further enlarge the termination edges of the W and S atoms, which explains why the size of WS_2_ crystal increases with the increase in gas velocity [34]. However, when the carrier gas velocity reaches as high as 200 sccm (Fig. 2c), the size of the WS_2_ crystals decreases rather than continue to increase. The reason is that when the velocity of the carrier gas is too fast, many S atoms flow out of the tube with the carrier gas before they can effectively react with the WO_3_ molecules around the substrates. Besides, too fast gas velocity will take away the heat on the substrate surface, resulting in a heat loss in the growth temperature.

Furthermore, the COMSOL simulations of gas velocity distribution for the front opening quartz at 100, 150, 200 sccm, respectively, were performed to study the mechanism of the influence of different gas velocities on the growth of WS_2_. As shown in Fig. 2d–f, the distribution of gas below the substrates increases significantly with the increase in gas velocity. Meanwhile, the temperature field at a fixed 950 °C was also considered and its distribution was obtained, as depicted in Fig. 2g–i. It is found that the temperature of the substrate is varied at different gas velocities. The higher the gas velocity, the lower the temperature of the substrate. Apparently, it is due to the heat conduction effect. When the gas flows through the substrates, it takes away part of the heat from the substrates. Figure 2j clearly depicts the temperature values along the middle line of the three sapphire substrates at gas velocities of 100, 150, 200 sccm. It is found that the gas velocity has a significant influence on the surface temperature of sapphire substrates. For a fixed gas velocity, the temperature of the sapphire substrate upstream is always lower than that downstream. Since the higher the gas velocity is, the more heat is taken away from the substrates, the larger temperature difference between the sapphire substrates upstream and downstream is. And the average temperature differences are 935, 901, 861 °C for gas velocity of 100, 150, 200 sccm, respectively. By taking a comprehensive consideration, 150 sccm is chosen as the optimized gas velocity.

**Fig. 3:**
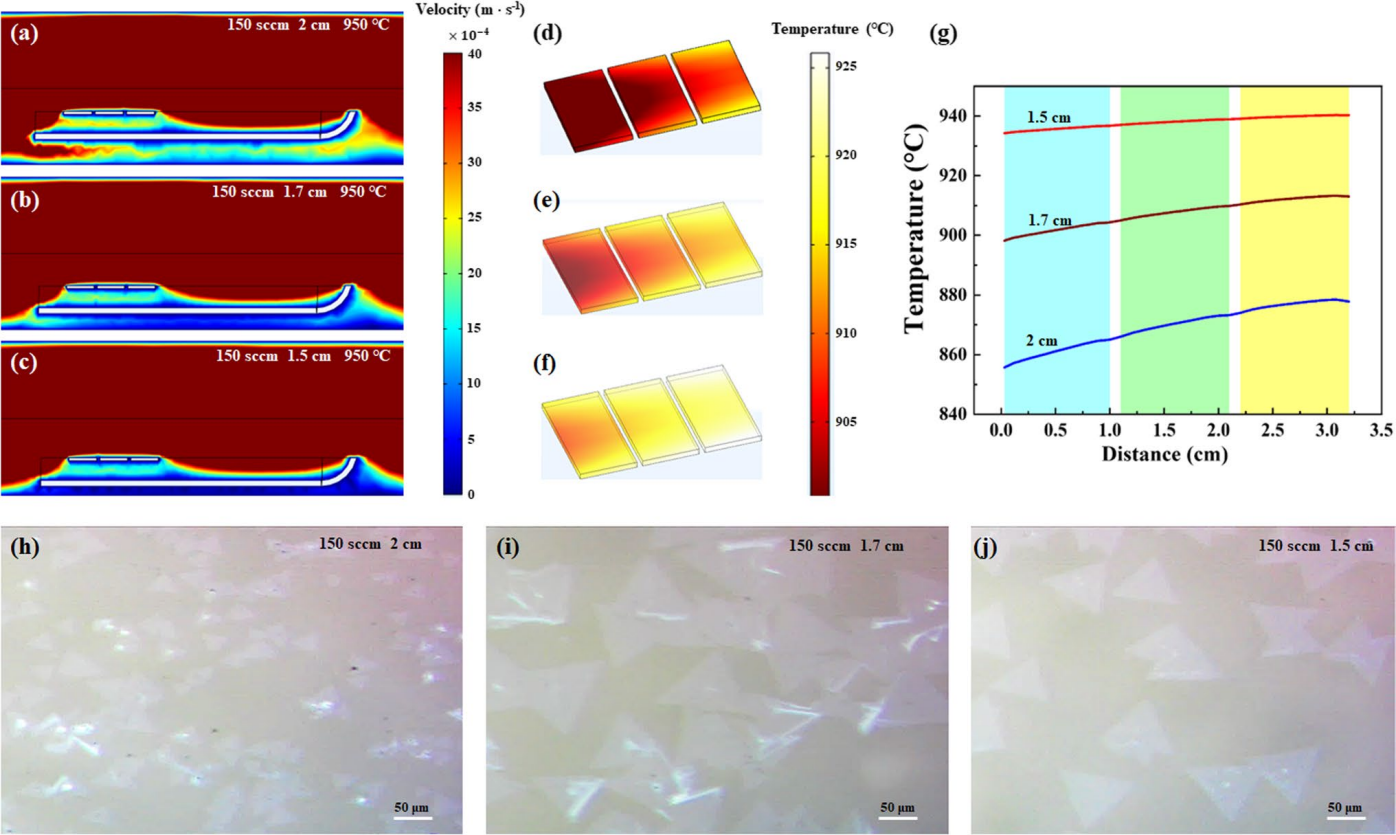
The COMSOL simulation of **a**–**c** gas and **d**–**f** temperature distribution for substrate with height of 1.5, 1.7 and 2.0 cm, respectively, away from the bottom of tube, with fixed gas velocity of 150 sccm and temperature of 950 °C. **g** The extracted temperature curves along the middle line of the three sapphire substrates. **h**–**j** The corresponding optical images of WS_2_ grown on sapphire substrates

Except that the gas velocity can affect the gas distribution around substrates, the height of the substrates located in the tube also influences the gas distribution. To control the height of the substrates located inside the tube, the front opening quartz boats are placed on a flat quartz with different widths of 3, 3.5 and 4 cm (Fig. S2), which corresponds to sapphire substrate heights of 1.5, 1.7 and 2 cm, respectively, away from the bottom of tube. From the simulation results about the effect of the sapphire substrate’s height on the gas velocity distribution inside the tube (Fig. 3a–c), the higher the position of the substrate is inside the tube, the more the gas distributes below the substrates. As Ar enters the furnace from the center of front end of the tube, the gas flows fastest around the central axis of the tube. As a result, when the front opening quartz boat gets closer to the center axis of the tube, the gas distribution below the sapphire substrate becomes larger. Hence, the influence of the height of the substrate inside the tube can’t simply be ignored. In addition, the temperature distributions for the three heights are depicted in Fig. 3d–f. A similar trend emerges, i.e., more heat is taken away from the substrate when the gas is higher below the substrate near the center axis of the tube. The temperature curves along the middle line of the three sapphire substrates are plotted in Fig. 3g. It further demonstrated that the height of sapphire substrates away from the tube bottom affects not only the gas distribution below the sapphire substrates, but also the temperature on the surface of the sapphire substrates. And the average temperature differences between the sapphire substrates upstream and downstream are 910, 901, 892 °C for heights of 1.5, 1.7, 2.0 cm, respectively. Figure 3h–j illustrates the morphologies of as-grown WS_2_ on the sapphire substrates with height of 1.5, 1.7 and 2.0 cm away from the bottom of the tube, respectively, when the temperature is fixed at 950 °C and the gas velocity is set to 150 sccm. There is also a significant change in the number and size of as-grown WS_2_ on the substrates. The WS_2_ flakes that grown on the sapphire with height of 1.7 cm exhibit larger size and more partially coalescent regions compared to the samples with heights of 1.5 and 2.0 cm. Hence, 1.7 cm is chosen as the optimized height by comprehensive consideration.

**Fig. 4:**
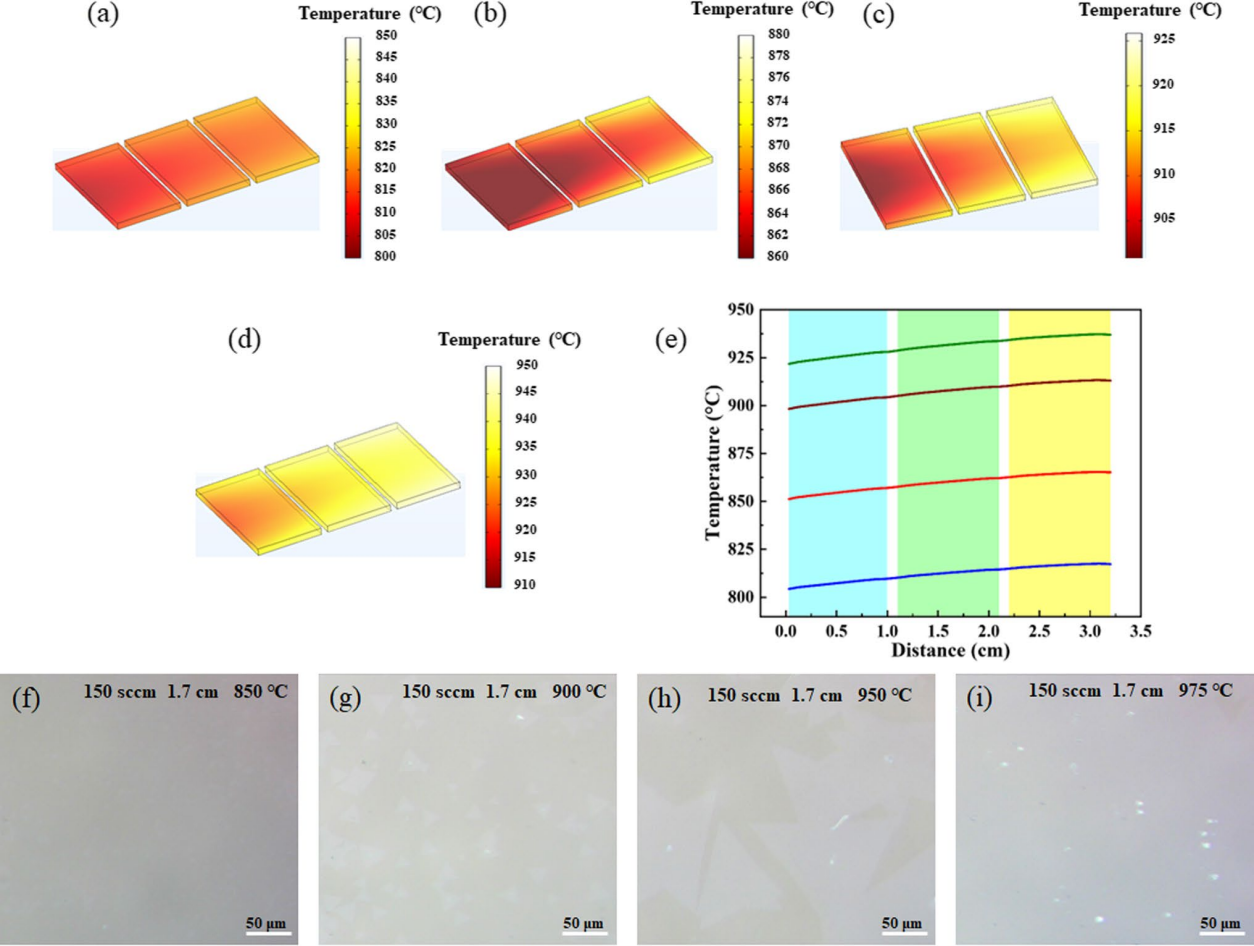
**a**–**d** COMSOL simulation of temperature distribution around the three sapphire substrates in the front opening quartz at 850, 900, 950, and 975 °C, respectively. **e** The extracted temperature curves along the middle line of the three sapphire substrates. **f**–**i** The corresponding optical images of WS_2_ grown on sapphire substrates

As known, the growth temperature can directly affect the reaction kinetic energy of W and S atoms, which in turn influences the domain size and growth rate of WS_2_ [35]. The temperature field simulations were performed with a gas velocity of 150 sccm and a height of 1.7 cm of the sapphire substrate from the tube bottom. As illustrated in Fig. 4a–d, with the increase in set temperature, the temperature of sapphire substrate also increases and presents inhomogeneous distribution. Combined with the curve plotted in Fig. 4e, the temperature of the sapphire substrate upstream is approximately 10 °C lower than that downstream due to the effect of gas flow. Besides, it can be found that the actual average temperature of sapphire substrate is much lower than the set value, with a difference between them of 43, 46, 49, and 51 °C at 850, 900, 950, and 975 °C, respectively. It has been reported that the higher the temperature is, the higher the sublimation rate of WO_3_ powder is, and the faster the growth rate on the substrates is [36]. At the growth temperature of 850 °C, the sapphire substrate has almost no triangular WS_2_ flakes, only with small and irregular nuclei (Fig. 4f), because the practical temperature of the substrate is 807 °C, which is lower than 850 °C. In this case, the temperature is too low to make WO_3_ vapor sublimate adequately, resulting in an insufficiency of the W source below the substrate. When the growth temperature is 900 °C, a few triangular WS_2_ flakes with size of about 24 μm appear alone (Fig. 4g). As the temperature increases to 950 °C, the domains become larger greatly and merge into large scale one (Fig. 4h). Considering the use of the front opening quartz boat, more gas is distributed around the substrate and it takes away part of the heat from the substrates. To compensate for the temperature taken away by the gas, the growth temperature is further increased to 975 °C. As a result, large-area and continuous monolayer WS_2_ film with a uniform surface topography and high coverage ratio was finally achieved on the substrates of sapphire (Fig. 4i). More OM images of continuous monolayer WS_2_ films are shown in Fig. S3. In a word, by carefully controlling the above growth parameters, large-area continuous WS_2_ films can be prepared and served as a potential candidate material for the electronic devices, especially for flexible and wearable sensors.

**Fig. 5:**
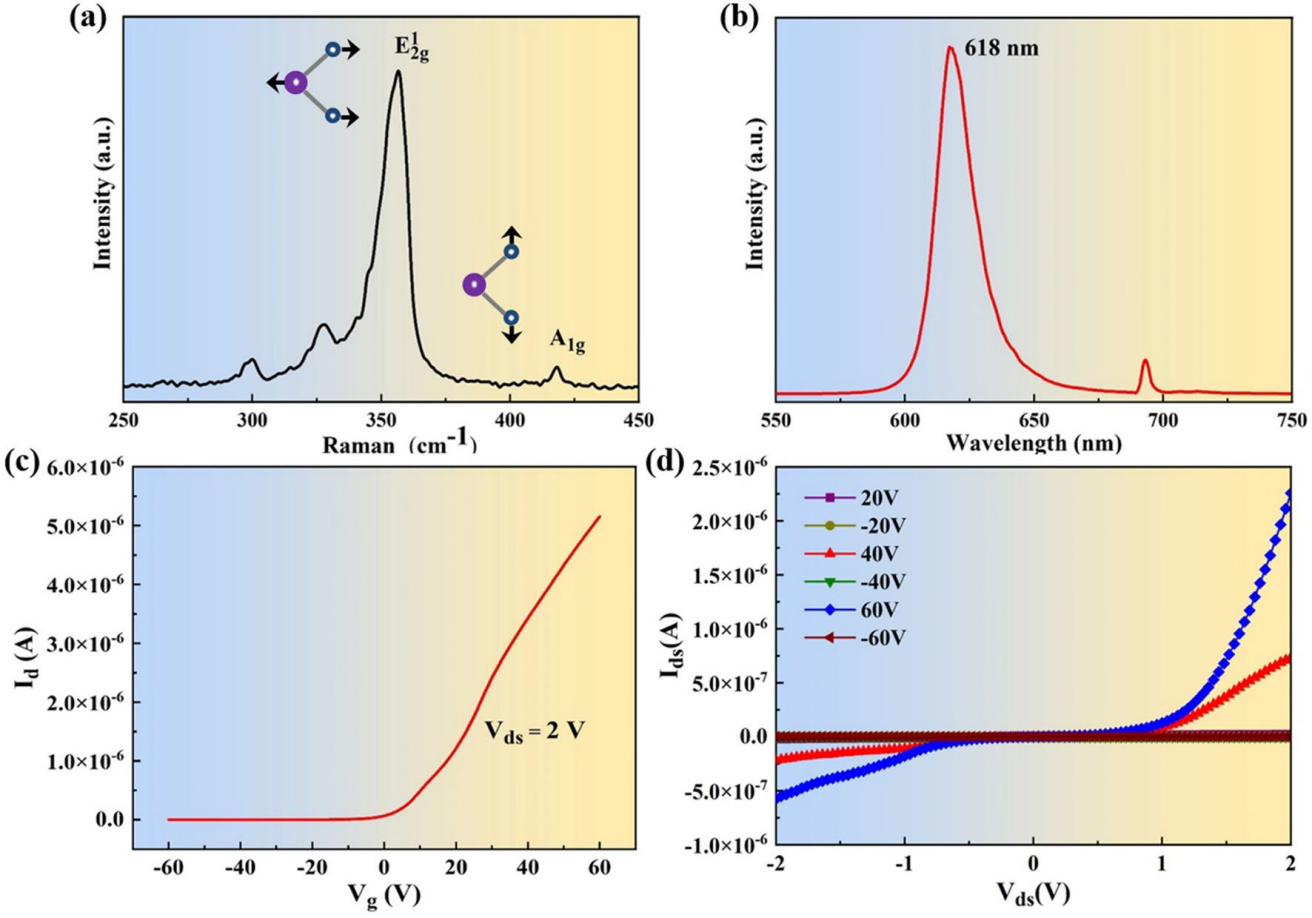
**a**–**b** Raman and PL spectra of as-grown WS_2_ film. **c**–**d** Transfer and output characteristics of the monolayer WS_2_ FET

To verify the quality and homogeneity of the as-grown WS_2_ films, large-scale and continuous films were selected for characterization. Raman and PL spectra measurements excited by 532 nm laser were performed on the samples to determine the properties of the monolayers. Figure 5a shows the in-plane $(E_{{2{\text{g}}}}^{1} )$ and out-of-plane (*A*_1g_) phonon modes of the as-grown WS_2_ films, which are located at 354.4 and 416.4 cm^−1^, respectively. The difference between them is 62 cm^−1^ that agrees with others’ reports for chemically synthesized monolayers WS_2_ [37, 38]. Figure 5b displays the PL spectrum of the as-grown WS_2_ films, which shows a strong PL peak at 618 nm (2.006 eV) assigned to the neutral exciton. PL intensity mapping around the peak and Raman intensity mapping at $(E_{{2{\text{g}}}}^{1} )$ over a large area of 200 × 200 μm^2^ of the continuous WS_2_ film are exhibited in Fig. S4. The atomic force microscope (AFM) image of as-transferred continuous WS_2_ film on SiO_2_/Si with a scratch is presented in Fig. S5, indicating a height of 0.8 nm thickness of WS_2_ monolayer. These observations confirm the monolayer and continuous nature of our CVD WS_2_ films [39].

Furthermore, to investigate the electrical property of as-grown WS_2_, the films were transferred from sapphire to SiO_2_/Si by wet transfer procedure similar to that reported by Bao et al. [40], as illustrated in Fig. S6. Then, transistors with Cr/Au electrode contacts were further fabricated on the transferred samples by laser direct writing lithography, followed by thermal evaporation deposition and removal of LOR/S1818 photoresist with acetone. The transfer characteristic curve of the as-fabricated transistors was measured and is plotted in Fig. 5c. It can be seen that the neutral point of the transfer curve locates closely to zero gate voltage, demonstrating the low intrinsic doping level in the transferred monolayer WS_2_ [41]. Additionally, the fabricated WS_2_ transistor devices are typically turned on at positive gate voltage, revealing that the grown monolayer WS_2_ is an *n*-type semiconducting material. Furthermore, the field-effect mobility of the device is extracted by

2 $$\mu = \frac{{dI_{{\text{d}}} }}{{dV_{{\text{g}}} }}\frac{L}{W}\frac{1}{{C_{i} }}\frac{1}{{V_{{\text{d}}} }}$$

where *I*_d_, *V*_g_, d*I*_d_/d*V*_g_, *L* and *W* are the drain current, gate voltage, slope, channel length, and width, respectively. *C*_*i*_ is the capacitance between the channel and back gate, estimated as ∼1.2 F/cm^2^ per unit area (*C*_*i*_ = *ε*_0_*ε*_r_/*d*, where *ε*_*0*_ is the permittivity of free space, *ε*_*r*_ = 3.9 and *d* = 300 nm) [42]. As a result, the electron mobility of the as-grown monolayer WS_2_ is calculated to be 3.76 cm^2^V^−1^ s^−1^ with a current modulation *I*_on_/*I*_off_ of ∼10^6^. It has been reported that electrons can be trapped by sulfur vacancies that act as charge scattering centers and reduce the electron mobility [43], and in addition, grain boundaries in polycrystalline material can lead to electron scattering, and higher growth temperatures (> 900 °C) can introduce high density of sulfur vacancies, which may be the reasons why the electron mobility is not very high for our samples. Figure 5d illustrates the output characteristic curve of the as-fabricated transistor at gate voltage *V*_bg_ ranging from − 60 to + 60 V in steps of 20 V. The nonlinear output characteristic indicates the existence of Schottky barrier between the Cr/Au metal contact and WS_2_ flake at the source and drain parts [19]. The expected higher mobility can be achieved in future work by introducing a high-k dielectric gate and optimizing the contact electrodes [44, 45].

**Fig. 6:**
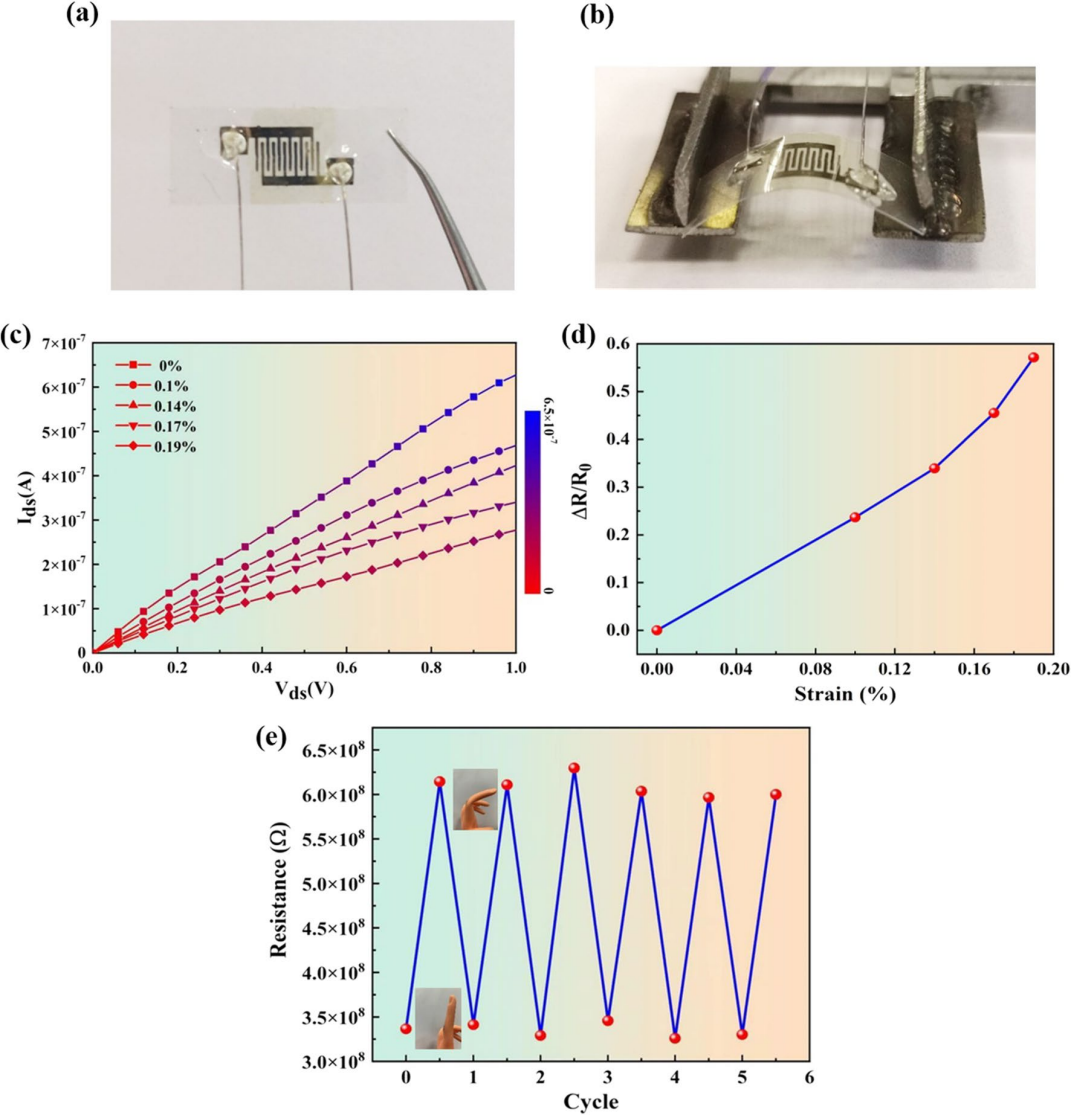
**a**–**b** Pictures of as-fabricated and bending WS_2_/PEN strain sensor, respectively. **c** I–V characteristic curves for the flexible WS_2_/PEN strain sensor under different applied strain. **d** Change rate of resistance of WS_2_/PEN strain sensor with increased applied strain from 0 to 0.19%. The GF can be calculated as ~ 306. **e** The resistance change response to repeating prosthetic finger bending. Insets are the flexible WS_2_/PEN sensor attached on the prosthetic finger to detect its bending condition

To verify the flexibility and sensitivity to strain of the as-grown monolayer WS_2_, [46–48], a transparent strain sensor based on WS_2_ film was fabricated on polyethylene naphthalate (PEN) substrate. Considering that the surface of the PEN substrate is hydrophobic, its surface first needed to be modified to be hydrophilic using a UV ozone cleaner for 40 min. Microelectronic flexible printer with silver ink material was used to print the interdigital electrodes on the pretreated PEN substrate. Also similar to the transfer method of Bao et al. [40] (as illustrated in Fig. S7), the WS_2_ films were transferred to the PEN substrate with pre-printed interdigital electrodes, and the final as-fabricated WS_2_/PEN strain sensor obtained is shown in Fig. 6a. The high-magnification OM images of the transferred WS_2_ films on PEN with electrodes are illustrated in Fig. S8, demonstrating that the WS_2_ films are successfully transferred onto the electrodes. In order to apply different strain conditions on the flexible WS_2_/PEN sensor, an instrument based on the modified Vernier caliper was prepared, as shown in Fig. 6b. Similarly, the applied strain can be calculated by the following equation [49, 50]

3 $$\varepsilon = \varepsilon_{zz} = \mp 3\frac{a}{2l}\frac{{D_{\max } }}{l}\left( {1 - \frac{{Z_{0} }}{l}} \right)$$

where *a* is the thickness of PEN substrate, *l* is the length of the strain sensor, *D*_max_ is the lateral shift of the free end of substrate, and *Z*_0_ is the distance between the fixed edge and the WS_2_ flake. Figure 6c plots the I–V characteristic curves of the flexible WS_2_/PEN sensor at different applied strain (*ε* = 0%, 0.1%, 0.14%, 0.17%, and 0.19%). Apparently, the I–V characteristics of the flexible WS_2_/PEN sensor change regularly as the applied strain increases, indicating that it is very sensitive to the strain. When the PEN substrate is bent, the monolayer WS_2_ films are elongated simultaneously leading to an increase in resistance due to the increased distance and weakened covalent bonds between the two neighboring WS_2_ molecules [51]. The relative change of resistance ∆*R*/*R*_0_ for each applied strain under voltage of 1 V is extracted, as illustrated in Fig. 6d. Obviously, the relative change of resistance increased linearly with increasing applied strain. The calculated GF = (∆*R*/*R*_0_)/*ε* is about 306, which exhibits better sensitivity than that of the strain sensors based on 2D In_2_Se_3_ nanosheets (~ 237) [52], bilayer (~ 230) and bulk MoS_2_ (~ 200) [50], and is comparable to that of a nanographene strain sensor (~ 300) [53, 54]. Besides, Fig. 6e shows the stable cycle of the resistance change response to repeating prosthetic finger bending. When the prosthetic finger bends, the resistance increases, and when it is straightened, the resistance nearly decreases to the initial value. It demonstrates a stable capability of detecting the finger bending movement for people and human–computer interaction.

## Conclusion

In summary, the CVD method using the front opening quartz boat is demonstrated, by which large-area and continuous WS_2_ films on sapphire substrates can be achieved successfully. The COMSOL simulations reveal that the front opening quartz boat can greatly enhance sulfur vapor distribution below the face-down sapphire substrate. Moreover, the height of the substrate located inside the tube also affects sulfur vapor and temperature distribution around the substrate. Size and continuity of WS_2_ can be well controlled by changing the temperature, gas velocity and the height of the substrates located inside the tube. Finally, large-scale and continuous monolayer WS_2_ films was achieved when the temperature was set at 975 °C, gas velocity was 150 sccm, and the sapphire substrate height was 1.7 cm away from the bottom of the tube. The FET based on the as-grown monolayer WS_2_ shows a field-effect mobility of 3.76 cm^2^V^−1^ s^−1^ and current modulation *I*_on_/*I*_off_ of ∼10^6^. Besides, a flexible and stretchable WS_2_/PEN strain sensor was fabricated with a GF as high as 306 and well stability under multi-cycle operation. These findings provide a promising way to transform the basic properties of 2D materials into various wearable devices and show great potential applications in healthcare monitoring, e-skins, and human–computer interaction.

## Supplementary Information

Below is the link to the electronic supplementary material.

Supplementary file1 (DOCX 3217 KB)

## Data Availability

Not applicable.
