# Supplementary material for: CVD growth of large-area monolayer WS2 film on sapphire through tuning substrate environment and its application for high-sensitive strain sensor
Source: Discov Nano. 2023 Feb 16;18(1):13. doi: 10.1186/s11671-023-03782-z (PMC9935800; doi:10.1186/s11671-023-03782-z)
Supplement: Supplementary file 1 — Supplementary file1 (DOCX 3217 KB) [file 11671_2023_3782_MOESM1_ESM.docx]

**Supplementary material**

**CVD growth of large-area monolayer WS_2_ film on sapphire through tuning substrate environment and its application for high-sensitive strain sensor**

Weihuang Yang^a*^, Yuanbin Mu^a^, Xiangshuo Chen^a^, Ningjing Jin^a^, Jiahao Song^a^, Jiajun Chen^b^, Linxi Dong^a*^, Chaoran Liu^a^, Weipeng Xuan^a^, Changjie Zhou^c*^, Chunxiao Cong^b,d*^, Jingzhi Shang^e^, Silin He^e^, Gaofeng Wang^a^, Jing Li^f^

^a^ Engineering Research Center of Smart Microsensors and Microsystems, Ministry of Education, College of Electronics and Information, Hangzhou Dianzi University, Hangzhou 310018, China.

^b^ State Key Laboratory of ASIC and System, School of Information Science and Technology, Fudan University, Shanghai 200433, China.

^c^ Department of Physics, School of Science, Jimei University, Xiamen 361021, China.

^d^ High Tech Center for New Materials, Novel Devices and Cutting Edge Manufacturing, Yiwu Research Institute of Fudan University, Chengbei Road, Yiwu City, Zhejiang 322000, China.

^e^ Institute of Flexible Electronics (IFE), Northwestern Polytechnical University (NPU), 1 Dongxiang Road, Chang'an District, Xi'an 710129, China.

^f^ Collaborative Innovation Center for Optoelectronic Semiconductors and Efficient Devices, Pen-Tung Sah Institute of Micro-Nano Science and Technology, Xiamen 361005, China.

**COMSOL simulation details**

To investigate the influence of quartz boat on airflow and growth temperature, finite element analysis software (COMSOL) is selected for modelling and simulation in this study. Firstly, we need to select modules and solvers. The modules added in this study are temperature field and fluid field modules according to the actual needs of physical fields. In addition, since we only need to investigate the temperature and airflow distribution in the steady state of the system, it is sufficient to select the steady state solver. The second step is to build the model and set the material properties. Figure S1(a) shows the structural model of the growth system constructed in COMSOL simulation software, which mainly includes the carrier gas (Ar), the tube furnace (total length 120 cm, radius 2.75 cm, of which the length of the preheater tube and the main furnace tube are 25 and 55 cm, respectively), the quartz boat carrier (length 10 cm, radius 1.25 cm) and the sapphire substrates (length, width and thickness of 2 cm, 1 cm, and 0.1 cm, respectively). As labelled in the figure, the gas inlet and outlet are at the A and B ends, respectively. Since the tube furnace is a dual-temperature zone and part of it is in a room temperature environment, it needs to be divided into several parts and then add the temperature field separately. The quartz boat is located in the main furnace of the tube furnace at a distance of 21 cm from the port of the main furnace and at a height of 0.9 cm from the bottom of the furnace tube. The three substrates are located inside the quartz boat with a distance of 3 cm from the front end of the quartz boat. In the material property setting, the material of tube furnace and quartz boat is set to quartz crystal, the material of substrate is set to sapphire, and the carrier gas argon used in the system can be found in the Elements unit of the material library. The third step is to set the module parameters. For the fluid field, we mainly consider the inlet and outlet of the gas and the setting of the gas flow rate, where the gas inlet is selected as mass flow, whose type is standard flow rate (sccm) with a value of 150 sccm, the standard pressure is set to 3 atm, the standard temperature is 293.15 K, and the furnace tube is under atmospheric pressure. To simplify the model, we directly set the double dual-temperature zone furnace tube as the heat source, where the initial temperature value of the whole system is 293.15 K. The steady states of the preheater and the main furnace are 423.15 K and 1223.2 K, respectively, and Figure S1(b) shows the heat radiation surface of the setup. Next, the model needs to be meshed, which can be done automatically by COMSOL software, or manually meshed. Because of the irregular shape and the irregular contact of the model, we choose to segment the model for better calculation. Specifically, in the main furnace, quartz boat, and substrate models, a more refined grid is used for the calculation, while for other simpler parts of the model, a super-coarsened grid is directly adopted for fast calculation. Figure S1(c) shows the model after grid meshing. Finally, after the model is meshed, the calculation of the whole physical field model is performed, and then the corresponding calculation data table and model diagram can be achieved.


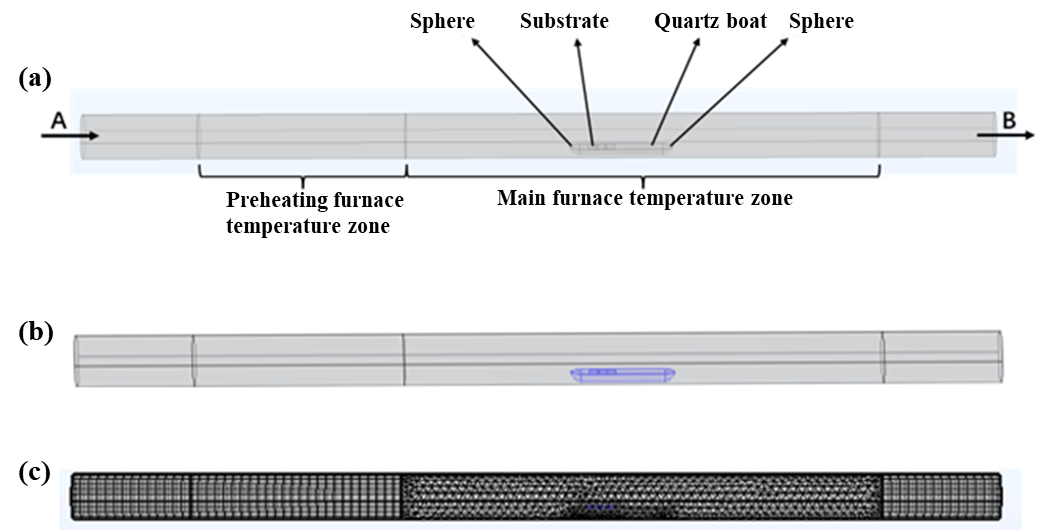


**Figure. S1.** (a) Structural model of the growth system constructed in COMSOL simulation software. (b) Thermal radiation surface set in the simulation software. (c) Model diagram after grid division.


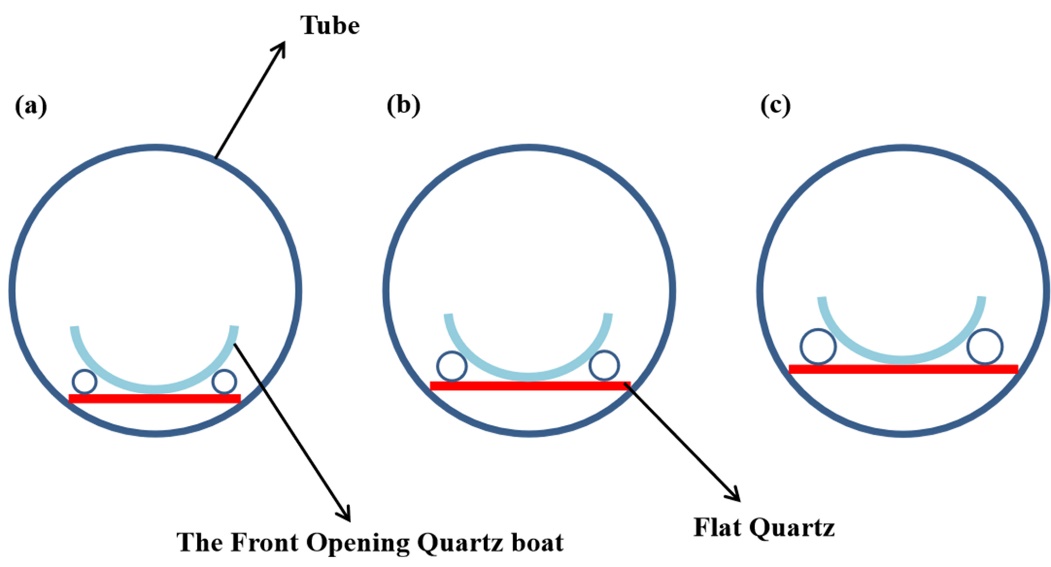


**Figure. S2.** Schematic of the front opening quartz boats that are placed on a flat quartz with different widths of 3, 3.5 and 4 cm, respectively.


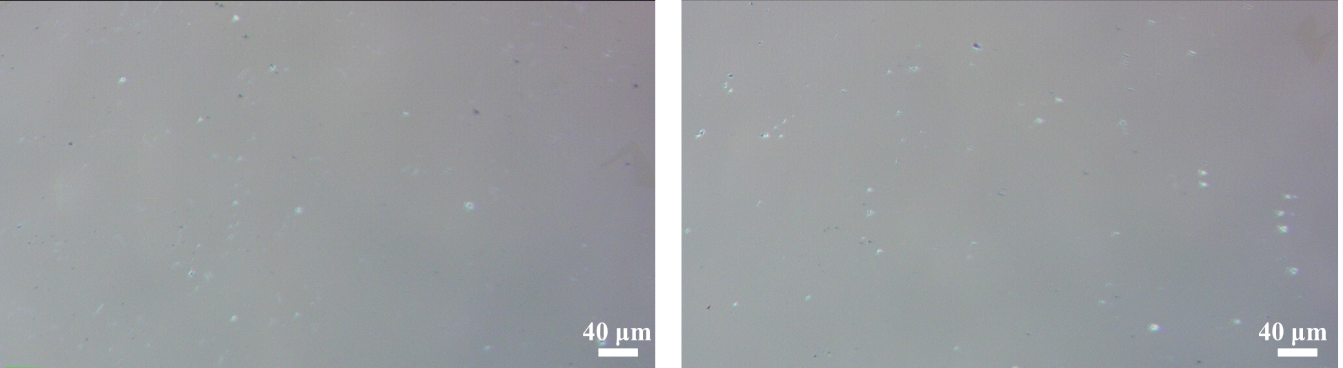


**Figure. S3.** The OM images of continuous monolayer WS_2_ films.


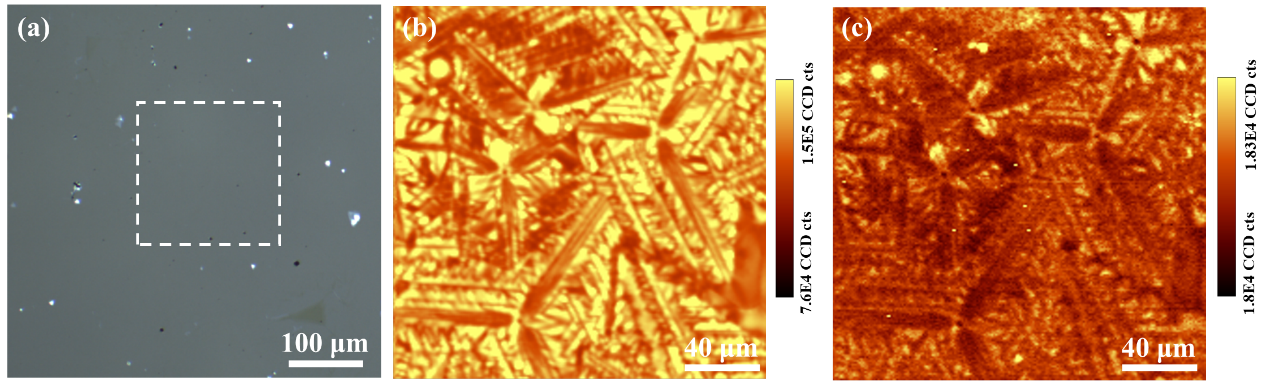


**Figure. S4.** Raman and PL spectra of the prepared sample over a large area of 200×200 μm^2^. (a) Optical image. (b) PL mapping. (c) Raman mapping.


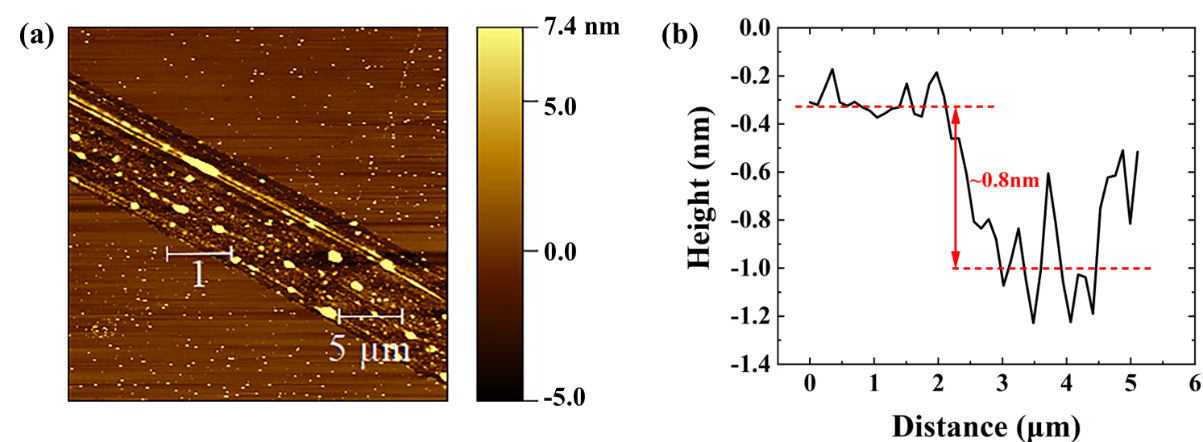


**Figure. S5.** (a) AFM image of as-transferred continuous monolayer WS_2_ film on SiO_2_/Si with a scratch and scan range of 30×30 μm. (b) Height distribution curve of the continuous WS_2_ film at symbol 1 in (a).


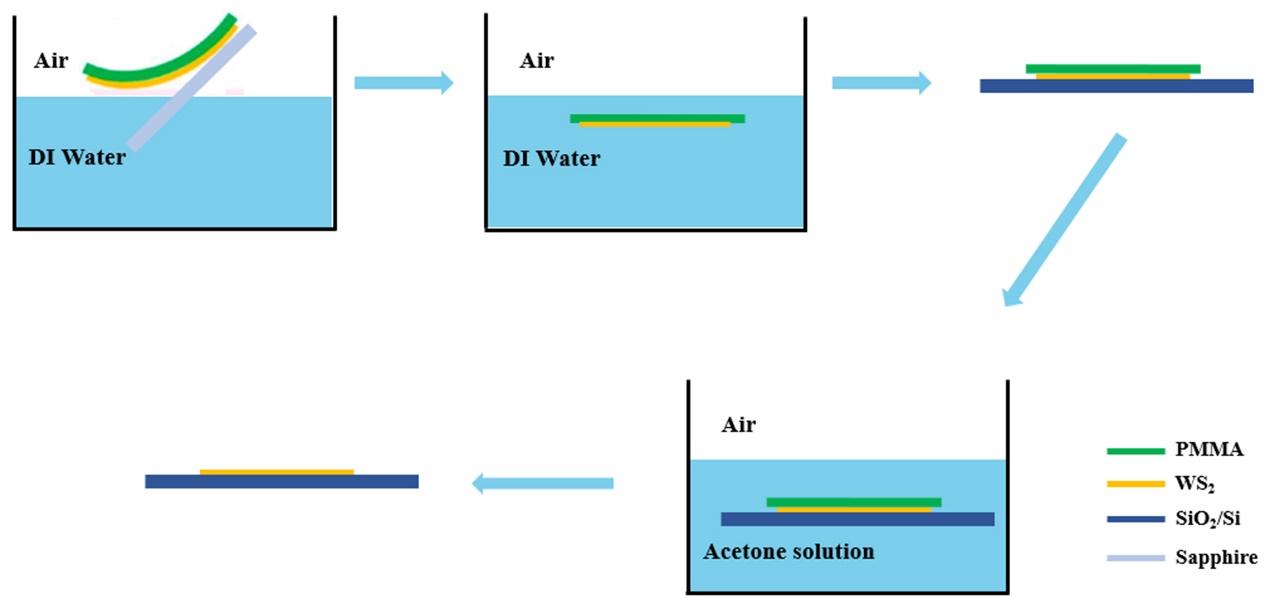


**Figure. S6.** Schematic of the sample transferred from sapphire to SiO_2_/Si substrate by wet transfer procedure.


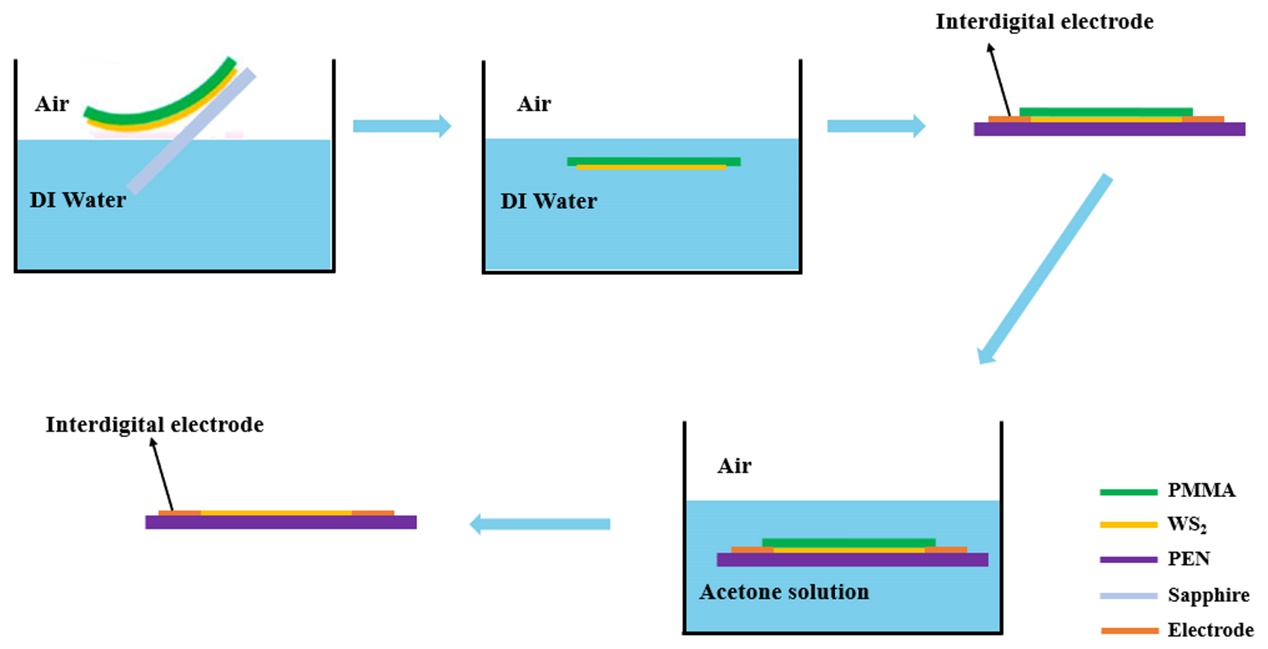


**Figure. S7.** Schematic of the sample transferred from sapphire to PEN substrate by wet transfer procedure.


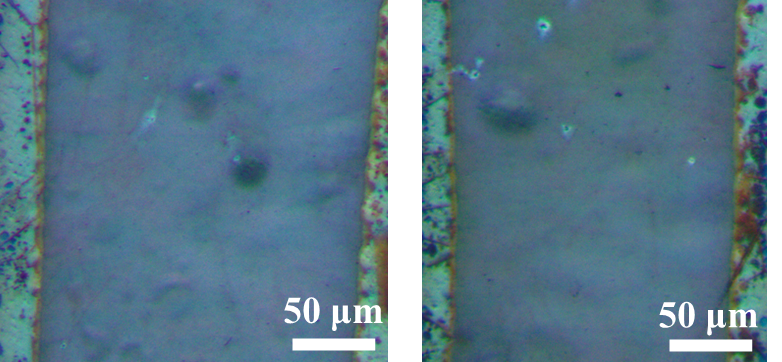


**Figure. S8.** Optical micrograph of inter-digital electrode on PEN. (a) Optical micrograph at 100x. (b) Optical micrograph at 200x.
